# Comparison of Bond Strength Between Three Types of Denture Teeth and the Acrylic Resin of a Complete Removable Denture Base: Comparison of Bond Strength Between Denture Teeth and the Acrylic Resin

**DOI:** 10.31661/gmj.v13iSP1.3616

**Published:** 2024-10-12

**Authors:** Shohreh Khalilzadeh, Soroush Etesami

**Affiliations:** ^1^ Department of Prosthodontics, School of Dentistry, Ahvaz Jundishapur University of Medical Sciences, Ahvaz, Iran; ^2^ Department of Periodontics, School of Dentistry, Ahvaz Jundishapur University of Medical Sciences, ahvaz, Iran

**Keywords:** Shear Bond Strength, Heat-Cured Denture Base Resin, Denture Teeth

## Abstract

**Introduction:**

To prepare a denture, the patient, dentist, and technician spend considerable time and money. The most common denture repair is the replacement and repair of detached teeth in a prosthesis. This study aimed to compare the bond strength between three different types of artificial teeth and a heat-cured acrylic denture base.

**Materials and Methods:**

In this in vitro experimental study, the shear bond strength of three groups of artificial teeth, including Apple & Glamor composite and B-Star nanocomposite, to a heat-cured acrylic resin denture base was compared. 10 samples were selected from each group. Samples were attached to the heat-cured resin. For bond strength assessment, the samples were placed in a universal testing machine and subjected to shear force at 1 mm/min speed, and the fracture load was recorded. Using SPSS 23 software and descriptive statistics, the mean force of fracture and the standard deviation of samples were calculated. One-way ANOVA and Tukey tests were used to compare the shear bond strength of the samples.

**Results:**

the mean shear bond strength of Apple composite teeth was recorded at 336 N. Also, for Glamor composite and B-Star nanocomposite denture teeth, the mean shear bond strength were recorded at 246 N and 154 N, respectively.

**Conclusion:**

The highest shear bond strength belong to Apple composite teeth and then to Glamor composite and B-Star nanocomposite denture teeth, respectively.

## Introduction

Complete removable dentures are used to restore the ability to chew, speak, and have esthetic restoration [1]. Denture teeth are a critical component in the construction of complete removable dentures, allowing for the restoration of chewing function, speech, and overall oral health [2]. The connection between the teeth and the denture base is considered an important factor for the longevity of a complete removable prosthesis. This affects patient comfort and quality of life indicators [3][4][5]. Although dental prostheses have made great progress in terms of materials and methods today, the separation of denture teeth from the denture base is still a problem. Separation of the teeth from the prosthesis base may occur due to the knockout, application of unfavorable occlusal forces, or inaccuracy in the laboratory steps of denture fabrication. Studies have shown that most repairs of removable prostheses are related to the separation of denture teeth from the denture base [6], so about 25-33% of the loss in removable prosthesis treatments is related to this process [7]. Separation of teeth from the denture base is more common in the anterior part. This problem can be due to the less contact of the teeth with the acrylic base denture in this area, as well as the angle of strength entering the anterior teeth during chewing [8]. Many factors, such as residual wax on the ridges on a tooth surface (ridge lap), inaccuracy in the use of separating materials during curing, the insufficient monomer used during curing, and the curing method used for denture base resin, have an effect on the bond between denture teeth and acrylic [9][10].

In various studies, the effect of contamination with wax, vaseline, and sodium alginate on the bond strength between the teeth and denture base has been investigated.It is observed that wax is the main contaminating factor and the main cause of failure in the bonding between teeth and acrylic surfaces [11]. In general, failure in bonding between denture teeth and denture base occurs in the form of adhesive and cohesive failure. Adhesive failure occurs when there is no sign of the denture base material on the ridge surface of the tooth after the failure, while failures are considered cohesive in which parts of the denture base material are visible on the ridge surface of the tooth after failure [8]. Several studies on the bonding of denture teeth to the base resin of the prosthesis show that generally, two processes are effective in building a successful bond between the denture teeth and dental acrylic resin: 1. The prosthetic acrylic resin must be bonded with denture teeth during polymerization. 2. The polymer network of dental acrylic resin must react with the polymer forming the denture tooth to create an interwoven polymer network [12]. The preparation of denture teeth can significantly impact the bond strength. Techniques such as applying monomer to the ridge surface, partially grinding to remove glaze, creating cavities, and modifying the ridge surface can either improve or compromise the bond between the teeth and the resin base of the prosthesis [13].

Ghafari Garabagh et al. (2019) found that Ivoclar teeth had an average bond strength of 392 MPa with monomer exposure and 337 MPa without [14]. Freitas de Andrade et al. (2018) reported that Kulzer Heraeus teeth had the highest bond strength (24.7 MPa) with light-cure acrylic, while Vipident teeth had the lowest (74.2 MPa) with thermoset acrylic [15]. Chittaranjan et al. (2013) found that sandblasted Endura teeth had the highest bond strength (87.6 MPa), while Rock Acry teeth had the lowest (61.3 MPa) [16]. Nematollahi et al. (2013) found that Ivoclar acrylic teeth had the highest bond strength (25.12 MPa) without cyclic loading, while Ivoclar composite teeth had the lowest (8.89 MPa) with cyclic loading [17]. Rostam Khani et al. (2012) reported that Ivoclar teeth had the highest tensile bond strength (206 Newtons), while Akradent teeth had the lowest (54 Newtons) [18]. Ghasemi et al. (2010) found that Apple teeth had the highest bond strength (1337 Newtons), while Glamor teeth had the weakest (880 Newtons) [12]. Naserkhaki et al. (2007) reported that Ivoclar Lichtenstein teeth had the highest bond strength (5.67 kg), while Marjan teeth had the weakest (3.50 kg) [19]. Saavedra et al. (2003) found that Vivadent teeth had higher bond strength with Ridge surface modifications and Triplex Hot acrylic resin [1]. Nejati Danesh et al. (2003) reported that Brilliant teeth had the highest bond strength with Acropars acrylic resin, while Super Newclar teeth had the weakest [6]. The bond strength between denture teeth and acrylic bases depends on the type of tooth and curing method used. Acrylic teeth have advantages over porcelain teeth, including reduced wear and destruction of occlusal surfaces, and chemical bonding with the prosthesis base [19]. It seems necessary to evaluate the characteristics of these products and check their bond strength to acrylic base dentures, considering the production of acrylic teeth and denture base resins in the country and the use of three types of denture teeth (Apple, Glamor, and Bay Star) by students in the School of Dentistry at Ahvaz Jundishapur University of Medical for fabricating complete removable dentures, and that the most common reason for repairing removable dentures is related to the separation of denture teeth from the acrylic base denture. This study aimed to provide dentists with sufficient information about the examined teeth so that it can be a reliable guideline for choosing the suitable tooth to determine the optimal treatment for edentulous patients and also help the manufacturer of this type of tooth improve its quality.

## Materials and Methods

This study is experimental research (laboratory) that was conducted at Ahvaz Jundishapur University of Medical Sciences (2018). In this study, three types of teeth —B-star, Glamor, and Apple—made by Ideal Makoo Co. (Tehran, Iran) were used. A total of 10 maxillary right central incisors were selected from each type of tooth, and thus the number of specimens was 30. Two millimeters more incisively than the deepest part of the ridge surface of the teeth was marked using a calibrated probe (Nordent, Illinois, United States), and a line with the same height was drawn around them. The ridge surface of the tooth was smoothed to the desired line, and thus the surface glaze was removed using a tungsten dental diamond bur (Teeskavan, Tehran, Iran) with a thickness of 1.2 mm [17]. Thermocycle thermal device (Vafaei industry, Tehran, Iran) TC-300 model was used to perform the thermocycle test. This device has two hot and cold water tanks with temperatures of 5 and 55 Celsius degree. In this device, specimens are placed in 5°C water for 30 seconds,removed from the cold water tank, and placed in the hot water tank at 55°C. The transfer of specimens between two tanks takes 10 seconds per cycle. In this study, all specimens were subjected tothermocycling 2500 times.

An Instron TC-KAP machine (Roell Zwick, Ulm, Germany) was used to apply force to the specimens and measure their bond strength to the acrylic base denture. This device is equipped with levers in different shapes to apply force to the specimens. The present study used a blade-form lever to simulate applied force on the teeth by the incisal edge of the opposite teeth. The initial force applied to the specimens was 5 Newtons. The device pressed the specimens at a speed of 1 mm/min until the time of failure. Then the breaking force of each specimen was recorded in Newtons.

The IBM SPSS 23 software (IBM Corp., Armonk, N.Y., USA) was used to mean across three or more groups of variables using one-way ANOVA, and Tukey's test was used to compare their pairwise differences. The current research lacked special ethical considerations because it was conducted in a laboratory and on dental materials. Kolmogorov-Smirnov and Shapiro-Wilk tests were used to check whether the breaking force of the specimens was statistically significant or not. All research variables had a normal distribution (P>0.05). P values of under 0.05 were considered significant.

## Results

**Figure-1:**
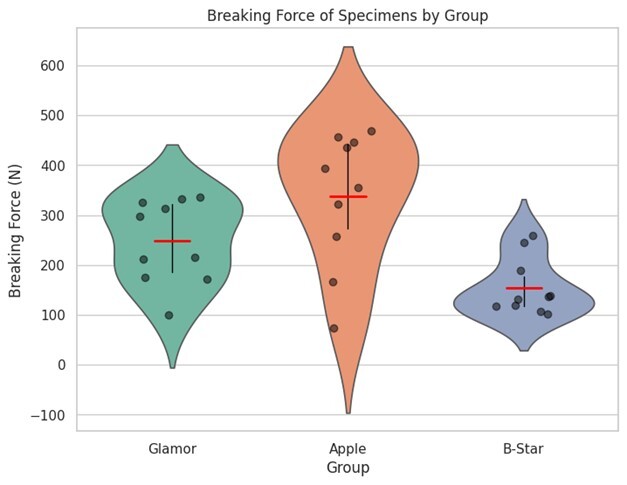
Figure 1. Breaking Force Distribution by Group; showing mean (red line), quartiles, and individual data points.

**Table:** 

**Group**	**Mean Breaking Force**	**Standard Deviation**	**n**	**Tukey’s Post-Hoc Comparison **	**p-value**	**Difference in Means**
Apple	336.91	133.0607	10	B star	<0.001	182.510
				Glamor	0.119	-88.940
B star	154.40	18.070	10	Apple	<0.001	-182.510
				Glamor	0.096	93.750
Glamor	246.43	26.41	10	Apple	0.119	88.940
				B star	0.096	-93.750

We examined the bond strength of Apple, Glamor, and B-Star denture teeth, with 10 samples of each type (Apple: 10, B-Star: 10, Glamor: 10). According to the results of the fracture strength test for all specimens in the Instron machine, their fracture force are presented in Figure-1.

The analysis of variance (ANOVA) revealed a significant difference in the mean breaking force of specimens across the three groups (Table-1): Apple, B-Star, and Glamor (P<0.001). The group means and standard deviations indicated that the Apple group had the highest mean breaking force (336.91 ± 133.06), followed by the Glamor group (246.43 ± 26.41), and then the B-Star group (154.40 ± 18.07). The large standard deviation in the Apple group suggests a high degree of variability in the breaking force values, which may be attributed to the inherent properties of the material or the testing conditions. In contrast, the Glamor and B-Star groups had relatively lower standard deviations, indicating a more consistent breaking force across the specimens.

The Tukey's post-hoc comparison test provided further insight into the pairwise differences between the groups. The results showed that the Apple group had a significantly higher mean breaking force compared to the B-Star group (P<0.001), with a difference in means of 182.51. This suggests that the Apple group had a substantially stronger breaking force than the B-Star group. However, the comparison between the Apple and Glamor groups revealed a non-significant difference (P=0.119), with a difference in means of -88.94. Similarly, the comparison between the B-Star and Glamor groups was also non-significant (P=0.096), with a difference in means of 93.75. These results suggest that while the Apple group had a significantly higher breaking force than the B-Star group, the differences between the Apple and Glamor groups, and between the B-Star and Glamor groups, were not statistically significant.

## Discussion

Removable dentures are used to restore the ability to chew, speak, and have esthetic restoration, and denture teeth are one of the main components of these prostheses. Improving the quality of denture teeth is essential considering the considerable time and money spent on making a complete denture, as well as the frequent and daily use of dentures by edentulous patients [20][21][22][23]. Comparing other Iranian specimens with these replacement teeth in terms of bonding strength with acrylic base dentures seems necessary with the entry of the new generation of denture teeth made by domestic companies such as B-Star Nano composite teeth into the market.

The study investigated the bond strength between three types of denture teeth (Apple, Glamor, and B Star) and heat-cured Ivoclar acrylic, commonly used in complete denture fabrication. The glaze on the ridge surface of specimens was removed with a diamond bur to increase monomer penetration and bond strength. Specimens were mounted in plaster and prepared to simulate the normal occlusion of the mouth. The same wax models were used to make the base attached to the teeth. The specimens were subjected to wax removal and acrylic curing, and the bond strength between acrylic denture teeth and heat-cured acrylics was evaluated. The study used Ivoclar heat-curing acrylic, which has higher bond strength than self-cure and light-cure acrylics due to increased methacrylate monomer penetration at high temperatures. The specimens were thermocycled 2500 times and evaluated using an Instron machine. The method used was similar to previous studies (Thean [24], Barpal [25], Clancy [26], and Cunningham [10]), where teeth were attached to the acrylic base denture from their base.

Researchers have found that physical and chemical changes can increase the bond strength between artificial teeth and denture bases. Physical changes include drilling holes and creating grooves on the ridge surface of artificial teeth. Chemical changes include impregnating the ridge surface with monomer, removing dental wax with boiling water and cleaning agents, washing with detergent powders, modifying polymer structure, and using resin cement [25][27][28][29]. In this study, cleaning materials and boiling water were used to clean the ridge surface, and the surface was exposed to monomer for 20 seconds before acrylic packing to increase bond strength. However, the effectiveness of these methods is outside the scope of this paper, as they were applied to all specimens. Harrison et al. found that factors like resin base type, teeth type, and copolymerization affect bond strength, and that thermosetting methods yield better bonds than self-polymerizing methods [30]. Therefore, a heat-cured acrylic denture base was used in this study.

Kawara et al. found that preparing teeth with monomers does not create enough bond strength [31], contradicting Speratley [32] and Barpal [25], but supporting Radford et al. [34] and Yamauchi [35]. However, most studies suggest that monomer use increases bond strength between teeth and acrylic base. This study found that B-Star teeth have the lowest bond strength, while Apple teeth have the highest bond strength to Ivoclar acrylic. Pairwise comparison showed that Apple teeth have significantly higher bond strength than B-Star teeth, with no significant difference between other groups. The difference in bond strength may be attributed to the structure of composite (Apple and Glamor) and nanocomposite (B-Star) teeth, as nanocomposite teeth have spherical silica nanofillers and a homogeneous polymer matrix [36], resulting in a shorter distance between particles and matrix, making bonding with acrylic more difficult.

Previous studies related to this research are reviewed. Ghaffari et al. (2019) investigated the bond strength of three types of denture teeth (Ivoclar acrylic, Apple composite, and B-Star nanocomposite) to heat-cure acrylic denture bases in Iran. The results showed that Apple composite teeth had significantly higher bond strength than B-Star nanocomposite teeth, consistent with the present study. However, the cooking method and type of thermosetting acrylic used were different, and thermocycling was not used [14]. Chittaranjan et al. (2013) studied the shear bond strength of three types of acrylic, composite, and nanocomposite denture teeth to an acrylic denture base. The study used a thermocycler and similar mounting and curing methods, but with aluminum cylinders instead of PVC pipes and heat-cured acrylic. The results showed that composite teeth had significantly higher bond strength than nanocomposite teeth, especially when sandblasted and impregnated with monomer, consistent with the present study [16]. Ghasemi et al. (2010) investigated the bonding strength of several types of multilithic artificial teeth (Glamor, Yaqut, Ivoclar, and Apple) to denture base resin. The study removed surface glaze from all specimens, used thermosetting acrylic, and exposed the ridge surface to monomer for 20 seconds before packing acrylic, similar to the present study. The results showed that Apple teeth had higher average bond strength than Glamor teeth, consistent with the present study [12].

Ghahremani et al. studied the effect of tooth preparation techniques on the tensile bond strength of Glamor composite denture teeth to denture base resin. The results showed that moisturizing the ridge surface with a monomer increased the bond strength, which is consistent with the present study's use of this method [37]. However, some studies had inconsistent results. Naserkhaki et al. (2007) found no significant difference in bond strength between Iranian artificial teeth and Ivoclar teeth, contradicting the present study. The difference in results may be due to the attachment technique, as Naserkhaki et al. attached the specimens to the acrylic base on the lingual surface, whereas the present study connected the teeth from the base to the denture base resin. Additionally, the type of acrylic used was different (heat-cured ACROPARS vs. heat-cured Ivoclar acrylic) [19]. Nematollahi et al. (2013) studied the bond strength of four types of denture teeth with Ivoclar acrylic and self-polymerizing denture bases. The results showed that the Ivoclar acrylic tooth had the highest bond strength, with no significant difference between Iranian Glamor and Marjan teeth. This contradicts the present study, which found a significant difference in bond strength between Glamor and Apple teeth. The difference in results may be due to the use of self-polymerizing acrylic in Nematollahi et al.'s study, whereas the present study used thermosetting acrylic [17].

## Conclusion

In general, the results show that the highest bond strength is related to the Apple artificial teeth with an average of 336 Newtons, and the lowest bond strength is the B-Star nanocomposite teeth with an average of 154 Newtons. The average bond strength of the Glamor teeth is 246 Newtons, which is between the Apple and B-Star groups. In the pairwise comparison of the groups, the bond strength of Apple teeth is significantly higher than that of B-Star teeth, while no significant difference is observed between the bond strengths of Apple and Glamor teeth with the acrylic denture base. Finally, there is no significant difference between the bond strengths of Glamor and B Star.

## Conflict of Intrest

None declared.

## References

[R1] Saavedra G, Neisser MP, Sinhoreti MAC, Machado C (2004). Evaluation of bond strength of denture teeth bonded to heat
polymerized acrylic resin denture bases. Braz J Oral Sci.

[R2] Adeyemi AA, Lyons MF, Cameron DA (2007). The acrylic tooth denture base bond: effect of mechanical
preparation and surface treatment. Eur J Prosthodont Rest Dent.

[R3] Critchlow SB, Ellis JS (2010). Prognostic indicators for conventional complete denture therapy:
a review of the literature. J Dent.

[R4] Preshaw PM, Walls AW, Jakubovics NS, Moynihan PJ, Jepson NJ, Loewy Z (2011). Association of removable partial denture use with oral and
systemic health. J Dent.

[R5] Emami E, Allison PJ, de Grandmont P, Rompre PH, Feine JS (2010). Better oral health related quality of life: type of prosthesis or psychological robustness. J Dent.

[R6] Nejati Danesh, Savabi O, Erfani M (2003). A comparative study of bonding strength between Iranian plastic
teeth and Acropars resin base material. J Dent Sch.

[R7] Vallittu PK, Lassila VP, Lappalainen R (1993). Evaluation of damage to removable dentures in two cities in
Finland. Acta Odontol Scand.

[R8] Yadav NS, Somkuwar S, Mishra SK, Hazari P, Chitumalla R, Pandey SK (2015). Evaluation of bond strength of acrylic teeth to denture base
using different polymerization techniques: A comparative study. JIOH.

[R9] Cunningham JL, Benington IC (1995). Bond strength variations of synthetic resin teeth in dentures. Int J Prosthodont.

[R10] Cunningham JL, Benington IC (1999). An investigation of the variables which may affect the bond
between plastic teeth and denture base resin. J Dent.

[R11] Barbosa DB, Barao VA, Monteiro DR, Compagnoni MA, Marra J (2008). Bond strength of denture teeth to acrylic resin: effect of
thermocycling and polymerisation methods. Gerodontology.

[R12] Ghasemi E, Mosharraf R, Eidi-Najafabadi A (2010). Evaluation of bond strength of four types of multilithic teeth to
acrylic denture base material. JIDA.

[R13] Mosharraf R, Mechanic N (2008). Comparison of the effects of four prebonding preparation methods
on the bond strength between a multilithic tooth and denture base resin. Dent Res J.

[R14] Gharebagh TG, Hamedirad F, Miruzadeh K (2019). A comparison of bond strength of three types of acrylic, composite, and nanocomposite artificial teeth to heat-cured acrylic denture base resin. Front Dent.

[R15] Andrade de, Brandt WC, Miranda ME, Vitti RP (2018). Effect of Thermocycling, Teeth, and Polymerization Methods on
Bond Strength Teeth-Denture Base. Int J Dent.

[R16] Chittaranjan B, Taruna M, Sudheer N, Patil NS (2013). Evaluation of shear bond strength of three different types of
artificial teeth to heat cure denture base resin: An invitro study. Indian J Dent Res.

[R17] Nematollahi F, Azizi N, Shahabi S, Ghahremani L, Asgari Z, Bagheri H (2013). Comparison effect of artificial tooth type and cyclic loading on
the bond strength to auto-polymerized acrylic denture base resins. JDM.

[R18] Rosthamkhani F, Gharehchahi J, Asadollahzadeh M, Zebarjad S, gharehchahi M (2012). Comparison of tensile strength of four kind of acrylic artificial
teeth to acrylic denture base in vitro. JMDS.

[R19] Naser Khaki, Ehsani S (2007). Comparing the bond strength of four kind of ideal-makoo
artificial teeth (Iran) and two leichtenstein & Italy ivoclar teeth with
prosthetic acrylic base. J Dent Sch.

[R20] Cunningham JL (1993). strength of denture teeth to acrylic bases.. J Dent.

[R21] Darbar UR, Hugget R, Harrison A (1994). Denture fracture: A survey. Br Dent J.

[R22] Jemt T (1991). Failures and complications in 391 consecutively inserted fixed
prosthesis supported by Branemark implants in edentulous jaws: A study of
treatment from the time of prosthesis placement to the first annual check up. Int J Oral Maxillofac Implants.

[R23] Cunningham JL, Benington IC (1994). Effect of an experimental cement on denture tooth bond. J Dent Res.

[R24] Thean HP, Chew CL, Goh KI (1996). Shear bond strength of denture teeth to base: a comparative
study. Quintessence Int.

[R25] Barpal D, Curtis DA, Finzen F, Perry J, Gansky SA (1998). Failure load of acrylic resin denture teeth bonded to high impact
acrylic resins. J Prosthet Dent.

[R26] Clancy JM, Hawkins LF, Keller JC, Boyer DB (1991). Bond strength and failure analysis of light-cured denture resins
bonded to denture teeth. J Prosthet Dent.

[R27] Wayne Caswell, Norling BK (1986). Comparative study of the bond strengths of three
abrasion-resistant plastic denture teeth bonded to a cross-linked and a
grafted, cross-linked denture base material. J Prosthet Dent.

[R28] Cunningham JL (2000). Shear bond strength of resin teeth to heat‐cured and light‐cured
denture base resin. J Oral Rehabil.

[R29] Reshadi S (2007). Comparison of bond strength between artificial resin teeth and
denture base through three method of mold preparation. J Mashhad Dent Sch.

[R30] Harrison A, Huggett R (1992). Effect of the curing cycle on residual monomer levels of acrylic
resin denture base polymers. J Dent.

[R31] Kawara M, Carter JM, Ogle RK, Johnson HH (1991). Bonding of plastic teeth to denture base resins. J Prosthet Dent.

[R32] Spratley M (1987). An investigation of the adhesion of acrylic resinteeth to
denture. J Prosthet Dent.

[R33] Morrow RM, Matvias FM, Windeler AS, Fuchs RJ (1978). Bonding of plastic teeth to two heat-curing denture base resins. J Prosthet Dent.

[R34] Radford D, Juszczyk A, Clark R (2014). The bond between acrylic resin denture teeth and the denture
base: recommendations for best practice. Br Dent J.

[R35] Yamauchi M, Iwahori M, Sakai M, Koda T, Kawano J, Maeno T (1989). Comparative bond strengths of plastic teeth to microwave-curing,
heatcuring and 4-META containing denture base resins. Gifu Shika Gakkai Zasshi.

[R36] Suzuki S (2004). Invitro wear of nano‐composite denture teeth. J Prosthodont.

[R37] Ghahramani L, Nokhbatolfoghahaei H, Shahabi S, Tamizi M, Fatemi M (2014). Effect of different teeth preparations on the tensile bond strength of composite artificial teeth to acrylic denture base. JDM.

